# Open source board based acoustofluidic transwells for reversible disruption of the blood–brain barrier for therapeutic delivery

**DOI:** 10.1186/s40824-023-00406-6

**Published:** 2023-07-15

**Authors:** Ke Wang, Chao Sun, Povilas Dumčius, Hongxin Zhang, Hanlin Liao, Zhenlin Wu, Liangfei Tian, Wang Peng, Yongqing Fu, Jun Wei, Meng Cai, Yi Zhong, Xiaoyu Li, Xin Yang, Min Cui

**Affiliations:** 1 State Key Laboratory of Agricultural Microbiology, College of Veterinary Medicine, Huazhong Agricultural University, Wuhan, 430070 People’s Republic of China; 2 Key Laboratory of Preventive Veterinary Medicine in Hubei Province, The Cooperative Innovation Center for Sustainable Pig Production, Wuhan, 430070 People’s Republic of China; 3 Key Laboratory of Development of Veterinary Diagnostic Products, Ministry of Agriculture of the People’s Republic of China, Wuhan, 430070 People’s Republic of China; 4 International Research Center for Animal Disease, Ministry of Science and Technology of the People’s Republic of China, Wuhan, 430070 People’s Republic of China; 5 School of Life Sciences, Northwestern Polytechnical University, Xi’an, 710072 People’s Republic of China; 6 Department of Electrical and Electronic Engineering, School of Engineering, Cardiff University, Cardiff, CF24 3AA UK; 7 School of Optoelectronic Engineering and Instrumentation Science, Dalian University of Technology, Dalian, 116023 People’s Republic of China; 8 Department of Biomedical Engineering, MOE Key Laboratory of Biomedical Engineering, Zhejiang University, Hangzhou, 310027 People’s Republic of China; 9 College of Engineering Huazhong Agricultural University, Wuhan, 430070 China; 10 Faculty of Engineering and Environment, Northumbria University, Newcastle Upon Tyne, NE1 8ST UK; 11 iRegene Therapeutics Co., Ltd, Wuhan, 430070 People’s Republic of China; 12 Department of Oncology, Hubei Cancer Hospital, Tongji Medical College, Huazhong University of Science and Technology, Wuhan, 430079 People’s Republic of China

**Keywords:** Acoustofluidic transwell, Blood–brain barrier, Surface acoustic wave, Human brain microvascular endothelial cells, Transendothelial electrical resistance

## Abstract

**Background:**

Blood–brain barrier (BBB) is a crucial but dynamic structure that functions as a gatekeeper for the central nervous system (CNS). Managing sufficient substances across the BBB is a major challenge, especially in the development of therapeutics for CNS disorders.

**Methods:**

To achieve an efficient, fast and safe strategy for BBB opening, an acoustofluidic transwell (AFT) was developed for reversible disruption of the BBB. The proposed AFT was consisted of a transwell insert where the BBB model was established, and a surface acoustic wave (SAW) transducer realized using open-source electronics based on printed circuit board techniques.

**Results:**

In the AFT device, the SAW produced acousto-mechanical stimulations to the BBB model resulting in decreased transendothelial electrical resistance in a dose dependent manner, indicating the disruption of the BBB. Moreover, SAW stimulation enhanced transendothelial permeability to sodium fluorescein and FITC-dextran with various molecular weight in the AFT device. Further study indicated BBB opening was mainly attributed to the apparent stretching of intercellular spaces. An in vivo study using a zebrafish model demonstrated SAW exposure promoted penetration of sodium fluorescein to the CNS.

**Conclusions:**

In summary, AFT effectively disrupts the BBB under the SAW stimulation, which is promising as a new drug delivery methodology for neurodegenerative diseases.

**Graphical Abstract:**

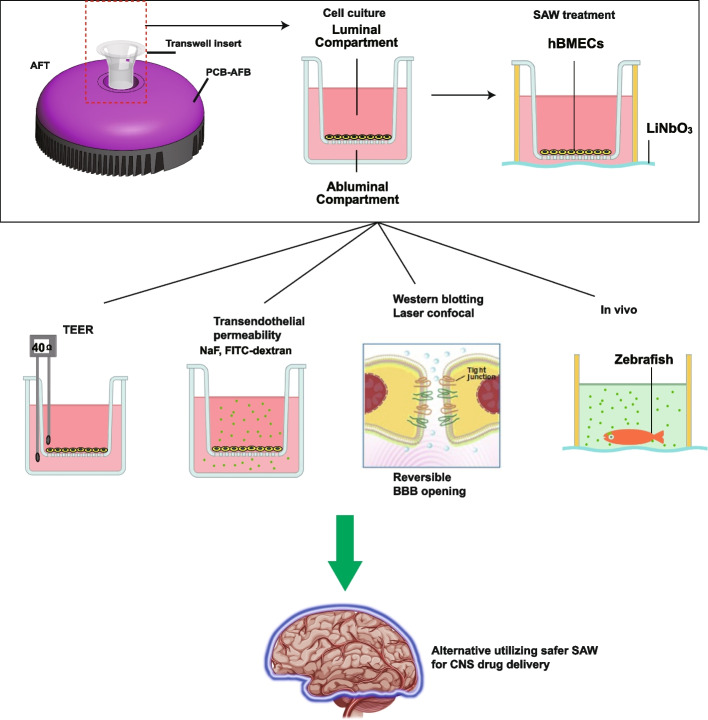

**Supplementary Information:**

The online version contains supplementary material available at 10.1186/s40824-023-00406-6.

## Introduction

**Fig. 1:**
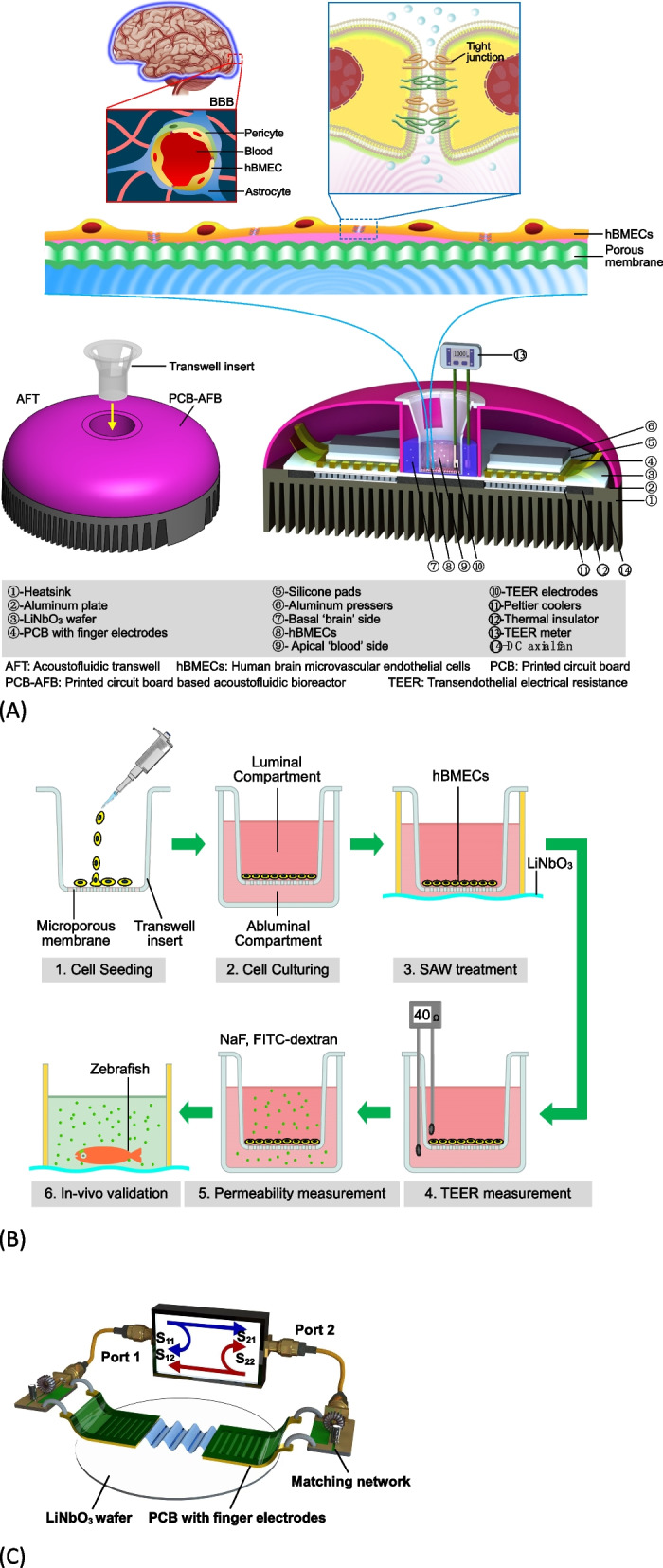
Schematic of the acoustofluidic transwell (AFT). **A** The AFT provides acoustic stimulation to the human brain microvascular endothelial cells (hBMECs) seeded on the transwell insert. The acoustic stimulation is produced by the surface acoustic wave (SAW) formed on the LiNbO_3_ wafer. The SAW is generated by using a printed-circuit board (PCB) based technique to configure the interdigital transducer (IDT). **B** The hBMECs are seeded on the transwell porous membrane forming luminal and abluminal compartments. The SAW exerts acoustic stimulation to the hBMECs to disrupt the tight junctions, which results in a decrease in the transendothelial electrical resistance (TEER) and increase in the transendothelial permeability. **C** The AFT is considered as a two-port network, as the two IDTs are constructed by pressing two PCB with finger electrodes onto the LiNbO_3_ wafer

The blood–brain barrier (BBB) is a multicellular vascular structure that separates the blood from the central nervous system (CNS) and regulates the delivery of important nutrients to the brain. The BBB presents at all levels of the vascular trees and acts as a vast and dynamic interface maintaining the cerebrovascular integrity to allow neurons to function properly [1]. As the core anatomical element of the BBB, brain microvascular endothelial cells (BMECs) have continuous intercellular tight junctions (TJs) and adhesions junctions (AJs) [2] (Fig. 1A). These TJs firmly seal the gaps between BMECs, thus resulting in a high transendothelial electrical resistance (TEER) of approximately 1.8 kΩ/cm^2^ [3], which limits paracellular permeability and regulates the diffusion of lipids and proteins. Occludin and Zonula occludens-1 (ZO-1) are typical TJ proteins, playing a critical role in maintenance of BBB functions, and their high-level expressions are correlated with high TEER and low permeability [4].

The BBB provides a tight control on the passage of nutrients from the vasculature into the extracellular fluid of the CNS, as well as shielding the brain from neurotoxic agents [5]. On the other hand, such substantial barrier presents one of the biggest challenges for drug delivery to the CNS due to low BBB penetration [6]. CNS diseases such as Alzheimer disease (AD), Parkinson disease (PD), glioblastoma, and rabies pose a huge threat to human health [7–9]. Because of TJs seal the BBB, macromolecular drugs in the plasma, such as neutralizing antibody against rabies virus and some glioblastoma, cannot target the virus or the tumor cells in the brain behind the BBB, unless the antibody is physically transported into the brain [10, 11]. Although many new drugs were developed in preclinical studies for treatments of Alzheimer disease, most of them clinically failed because of the low brain penetration, and only a few drugs with high brain penetration showed therapeutic efficacy, which clearly indicates that improving the drug delivery is the key for therapy of neurodegenerative disease [12]. However, drug delivery through the BBB requires reversible disruption of TJs to enable temporary paracellular transport routes between BMECs [13]. Therefore, it is crucial to recover the barrier function following entry of therapeutic agents into the brain to avoid the potential influx of unwanted molecules and immune cells leading to the neurotoxicity. BBB disruption has previously been observed in the symptoms-stage of AD [14], implying that the uncontrolled opening of the BBB accelerates the disease course.

Safe blood–brain barrier opening (BBBO) is critically important in drug delivery, which requires controllably reversible unzipping of the TJs. Current BBBO approaches can be generally categorized into chemical and mechanical ones. Among the mechanical ones, ultrasound can generate mechanical pressure wave with frequencies normally above a few kHz, up to a few MHz. This modality has been broadly used in anatomical imaging and hemodynamic measurement [15, 16]. Focused ultrasound generates and utilises microbubbles to disrupt the TJs via cavitation, leading to transient BBBO [4]. For example, ultrasound working at 220 kHz had been demonstrated to aid the BBBO and temporarily open BBB in patients with Parkinson disease and dementia, resulting in mild cognitive improvement [17]. Bevacizumab delivery assisted by focused ultrasound working at similar frequencies was also reported to significantly retard glioma progression with an increased median survival of 135% [18]. Despite the enhanced BBB permeability was reported, the microbubble cavitation induced BBBO is well-known to be unpredictable and even cause apparent damages to brain tissue [19]. One potential improvement when using the ultrasound to induce BBBO includes increasing the ultrasound frequency up to MHz level, so that the acoustic cavitation is minimal.

Surface acoustic waves (SAWs), with frequencies ranging from a few MHz to a few GHz, have been demonstrated in producing nanoscale surface vibrations with their wavelengths comparable to sizes of biological cells [20]. SAWs propagate along the surface of solid materials with most of their acoustic energy trapped near the surface. This characteristic provides great advantages of integrating micro-systems such as microfluidics on the surface of a SAW device, allowing an effective manipulation of bioparticles in an acoustofluidic system [21]. For example, SAW stimulation accelerated neural differentiation process of human embryonic stem cells with a great potential in shortening the time for cell therapy manufacturing [22]. Acoustic excitation working at MHz levels has also been demonstrated to effectively internalize molecules into cells while maintaining the integrity of both the therapeutic agents and the host cells [23]. The mechanism of cell transfection might be associated with temporal reorganization of the lipid structures in the cell membrane that allowed therapeutic molecules to be delivered [24], and acoustic streaming is supposed to facilitate this process [23]. Another SAW based device working at MHz ranges has been successfully demonstrated to increase the motility of human sperm by 15% [25]. SAW has also been applied to assemble patient-derived cell clusters to model tumour organoids, which can be implemented for personalised cancer adjuvant therapy [26].

To date, transwell system is one of the simplest and also most commonly seen in vitro BBB models. In this system, a single layer of BMECs is seeded on top of the upper semipermeable membrane, separated from the abluminal (apical) (Fig. 1B). For technical reasons to combine SAW device with in BBB, current rarely researches applying SAW for blood brain barrier opening. In this study, we activated the BBB model on the transwell system by applying a SAW with a frequency about 20 MHz. The proposed acoustofluidic transwell (AFT) acquired the advantage of our open-source acoustofluidic systems based on printed circuit board architecture [27–29], which has merits of rapid prototyping on-demand and versatile in combination other micro-systems. The AFT was operated under various SAW agitation dosages. The preliminary tests clearly demonstrated that the AFT is able to control the opening of the BBB with reversible disruption of TJs. The applicable SAW dosages were demonstrated to create temporary paracellular transport of substances with different molecular weights. The expression of Occludin and ZO-1 showed no significant changes, but intercellular space was enlarged after SAW administration. After the treatment of SAWs, the hBMECs were recovered with a restored TEER. Finally, we validated the BBBO using zebrafishes to demonstrate the permeation of sodium fluorescein into the brain tissue. Our results confirmed that the AFT is particularly useful in regulating the BBBO for drugs delivery across the blood–brain barrier.

## Materials and methods

### Working mechanism

The AFT provides fundamental functionalities of the BBB and facilitates delivery tests through the endothelial cells under the assistance of the SAWs, which were generated using a printed circuit board based acoustofluidic bioreactor (PCB-AFB) [22] and a transwell insert as shown in Fig. 1A. Human brain microvascular endothelial cells (hBMECs) were seeded as a single cell layer on the upper side of the porous membrane on the transwell insert as shown in the protocol described in the following session (Fig. 1B). During the treatment, the transwell insert was plugged into the socket on the PCB-AFB. The internal configuration of the AFT is displayed in Fig. 1A, which is detailed in the device fabrication session.

The whole process is illustrated in Fig. 1B. After seeding and culturing the hBMECs (Step 1 and 2), the transwell insert is plugged and submerged in the medium in the socket (Step 3), where the SAWs produced from both sides interact with the hBMECs through the porous membrane. The TJs between endothelial cells are affected, resulting in an enlarged gap between cells. Such ‘opening’ effect can be reflected by measuring the transendothelial electrical resistance (TEER) (Step 4). The TEER is a widely accepted quantitative technique to assess the integrity of TJ dynamics in cell culture models of endothelial monolayers [30–32]. The enlarged gaps allow pharmaceutical agents to penetrate through from the ‘blood’ side (luminal compartment) to the ‘brain’ side (abluminal compartment) (Step 5). The AFT works as a tool to control the BBBO without the need of any opening agents. The validation of the AFT is performed using a zebrafish model, where the zebrafish is administrated with an appropriate SAW dose to test the penetration of the BBB (Step 6).

### Fabrication of the Acoustofluidic Transwell (AFT)

The AFT is a highly accessible acoustofluidic device fabricated by a number of off-the-shelf components. Figure 1A shows all the components assembled the AFT and the inner architecture of the device. A rapid prototyping of the device is available on demand without the need of cleanroom manufacturing.

A heatsink (① in Fig. 1A, Farnell UK, order code: 1,850,032) was used as the base support for the AFT and responsible for heat dissipation. The heat exchange between the heatsink and the environment was accelerated by using a DC axial fan (⑭, Farnell UK, order code: 3,794,029) mounted under the heatsink (①). Two peltier coolers (⑪, Farnell UK, order code: 1,639,724) were mounted on the heatsink with their cold sides coupled to a customised aluminum plate (②) with a thickness of 1 mm. Heatsink grease (Farnell UK, order code: 1,663,190) was applied on both sides of the peltier coolers to improve the thermal coupling. Due to poor thermal conductivity of LiNbO_3_, a thermal conductive sheet with the thickness of 1 mm made by silicone (Farnell UK, order code: 1,893,451) was mounted on the top surface of the aluminum plate to couple any heat from a LiNbO_3_ wafer (③, 128° Y-cut X-propagation, 3 inch). The interdigital transducer (IDT) function was generated by pressing and finger electrodes patterned on a thin film printed circuit board (PCB) onto a LiNbO_3_ wafer (④). The PCB design file was submitted to a PCB manufacturer (circuitfly.com) for fabrication. The PCB substrate was a flexible polyester film with a thickness of 70 μm, on which 40 pairs of finger electrodes with a period of 200 μm and an aperture size of 2 cm were patterned by depositing two metal layers, nickel/gold (2 μm/30 nm thick). To firmly couple the PCB to the LiNbO_3_ wafer, silicone pads (⑤) and aluminum pressors (⑥) were stacked onto the PCB using additional mechanical structure (not shown in Fig. 1A) to enforce the contact between the finger electrodes and the wafer.

Amplified radio frequency signals were fed into both the IDT and converted into SAWs propagating on the surface of the LiNbO_3_ wafer. A compartment of PLA plastic was 3D printed for accommodating the transwell insert and storing medium for the ‘brain’ side (⑦). The volume of the ‘brain’ side was 12 mL for fully submerging the transwell insert, which contacted with the LiNbO_3_ wafer for coupling acoustic waves to the hBMECs (⑧) on the membrane. The TJs on the hBMECs were disrupted by the acoustic energy, whose opening can be quantified by measuring the electrical resistance across the hBMEC monolayer separating the ‘blood’ side (⑨) to the ‘brain’ side (⑦). The TEER meter (⑬) read the resistance between its two electrodes (⑩), which indicate the integrity and permeability of the hBMEC monolayer.

### Device characterization

The width and spacing of the finger electrodes on the PCB are key parameters determining the working frequency of the AFT. After manufacturing, the integrity and accuracy of the finger electrodes were checked under a measuring microscope (Soptop, CX40M). The AFT consisting of two IDTs was treated as a two-port network whose S-parameters were measured using a vector network analyzer (VNA, E5061B, Keysight, US). As shown in Fig. 1C, the reflection coefficient and the insertion loss are monitored to indicate the assembly quality and alignment of the two IDTs, respectively. Measuring the reflection coefficient is essential to inform the process of impedance matching for the IDTs [33]. The dip frequency read from the reflection coefficient S_11_ / S_22_ was registered as the working frequency of the AFT. A temperature control was used to maintain the temperature in the transwell insert under different SAW doses. The choice of these SAW doses was demonstrated by measuring the microparticles streaming velocity using an optical the microscope.

### Device modelling

To evaluate the acoustofluidic field in the AFT, a 2D model of the device was built in COMSOL Multiphysics 5.5. (COMSOL Inc., USA). Ten pairs of IDTs were modelled on the LiNbO_3_ wafer. The transwell and support beams were modelled as linear elastic materials made of PLA plastic, and the bottom of the transwell was selected as porous polycarbonate. The acoustic pressures generated by leaky SAWs were obained under the Thermoviscous Acoustics module in the frequency domain. Governed by incompressible Navier–Stokes equations, the laminar flow module was used to calculate the drag and acoustophoretic forces as well as liquid displacement effects due to the previously calculated acoustic pressure. Forces obtained in the liquid were coupled with a time domain study and particle tracing module, to observe cell barrier deformation.

### Cell culture and transwell BBB model

hBMECs were maintained in RPMI 1640 medium supplemented with 10% (vol/vol) fetal bovine serum (FBS, Gibco, Grand Island, NY), penicillin/streptomycin, endothelial cell growth supplement (L-glutamine, sodium pyruvate, amino acid, vitamin) as previously described with modification [34]. The in vitro BBB model was adapted from established one according to reported scheme [35, 36]. Briefly, a 6.5 mm transwell (Costar, Corning, USA) was coated with collagen IV, then the hBMECs were seeded in the transwell inserts with the population of 8 × 10^4^ cells. After the cells reach 100% confluent (24 h and 48 h), the transwell inserts were either left untreated or exposed to SAWs with various doses.

### TEER and transendothelial permeability

The TEER was measured with an EndOhm electrodes (World Precision Instruments, WPI, Sarasota) combined with Millicell ERS-2 resistance meter (Millipore, Billerica, MA) according to the manufacturer's instructions. For transendothelial permeability assay, 100 μg/ml NaF, 1 mg/ml FITC-dextran-10 kD (Sigma-Aldrich, St. Louis, MO), or 1 mg/ml FITC-dextran-70 kD (Sigma-Aldrich, St. Louis, MO) were added to the up chamber of transwell insert after SAW stimulations. After 5 min incubation, the fluorescence density of upper chamber and lower chamber were measured at excitation at 488 nm and emission at 530 nm.

### Cell viability detection

The confluent hBMECs were exposed to SAWs with various powers, then the CCK8 reagent (Biosharp Life Science, China) was added into each well. After 1 h incubation at a 37℃ incubator, the absorbance was detected with a microplate reader at a wavelength of 450 nm.

### Western blotting and Immunofluorescence

The hBMECs being exposed to the SAWs were harvested with RIPA buffer containing protease inhibitor. A BCA kit (Beyotime, China) was used to measure and adjust the protein concentrations. The cell lysis was mixed with loading buffer and incubated at boiling water for 10 min. Samples were then subjected to sodium dodecyl sulfate polyacrylamide gel electrophoresis. The proteins in gel were transferred to polyvinylidene difluoride membranes (Millipore). After blocking with 5% skim milk, the membrane was hybridized with anti-Occludin, ZO-1, or β-actin (Proteintech, China) antibody. The signal was amplified by conjugating to HRP-labeled second antibody, and results was observed with ECL chromogenic solution (Beyotime, China).

For immunofluorescence, the hBMECs were fixed with 4% paraformaldehyde. Cells were then blocked with 2% BSA, and anti-Occludin or anti-ZO-1 polyclonal antibody (Proteintech, China) was applied to mark the targeted proteins, followed by conjugation using Alexa Fluor 488 labeled second antibody. The images were captured on a laser confocal microscope (Leica SP8, Germany).

An Annexin V-AbFluor-647 Apoptosis Detection kit (Abbkine, KTA0004) was used for cell apoptosis detection according to the manufacturer's instructions. Briefly, the hBMECs were digested with 0.25% trypsin and then stained with Annexin V and PI reagent after various SAW treatment. Results were acquired in CytoFLEX (Beckman Coulter, Brea, CA) and analyzed with CytExpert Software (Beckman Coulter, Brea, CA).

Ten-day-old zebrafish (Danio rerio, 10 days post fertilization, transparent body) were kept in 1 mg/ml NaF water of AFT system, and exposed to SAWs under an input power of 37.8 dBm (about 6 W), while the control was sustained in 1 mg/ml NaF water. After exposed to the SAW agitation for 5 min, the zebrafish was washed in fresh water and the fluorescence in the brain was imaged using a fluorescence microscope (Olympus IXplore, Japan). After experiment, all zebrafish were euthanized by a full dose of tricaine methanesulfonate (Sigma) and safely disposed.

### Statistical analysis

Data of the study are showed as mean ± standard error of mean (SEM) unless otherwise stated. The significance was tested with two tailed t test, one-way or two-way analysis of variance (ANOVA) followed by Tukey’s post hoc tests with GraphPad Prism software (v7.0; GraphPad, La Jolla, CA). The results are the representative of at least two independent experiment which was detailed in the figure legends.

## Results

### Numerical simulation and characterization of the AFT

**Fig. 2:**
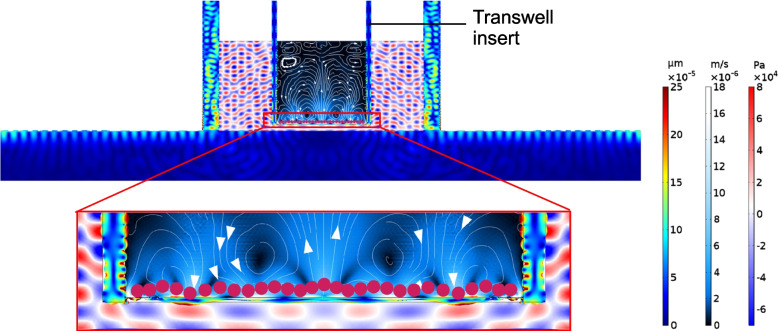
Numerical simulation of the acoustofluidic transwell (AFT). The SAW propagates on the surface of the substrate, inducing the leaky SAW in the transwell. The inset shows the leaky SAW coupling into the transwell insert, which produce pressure gradients in the medium

The acoustofluidic modelling of the AFT displays the SAWs to propagate through the porous membrane on the transwell insert and arrive the hBMECs. As the simulation result shown in Fig. 2, the SAW leaks into the medium underneath the porous membrane, which creates a first-order acoustic pressure field inside the abluminal compartment. The acoustic waves encounter with the porous membrane and convert into the membrane vibrations, exerting mechanical stimulation to the attached hBMECs. The TJ proteins between the hBMECs are considerably disrupted by the mechanical vibrations. The acoustic energy is then passed through the cells and finally absorbed in the luminal compartment inside the transwell insert, which produces intensive streaming to enhance the collisions between the pharmaceutical agents and the hBMECs.

**Fig. 3:**
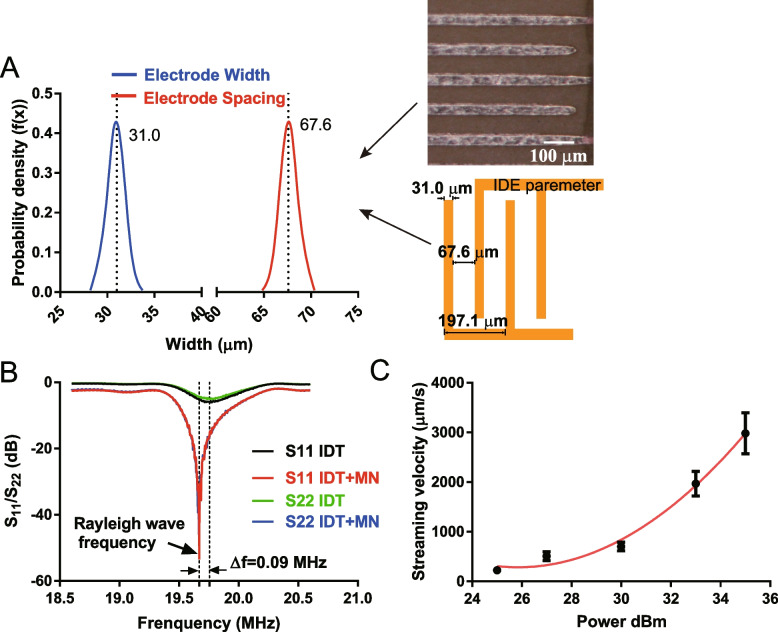
Characterisation of the acoustofluidic transwell (AFT). **A** The width and spacing of the interdigital electrodes patterned on the printed circuit board were found to be 31.0 ± 1.1 μm and 67.6 ± 0.9 μm, respectively, resulting in the wavelength of 197.1 ± 2.4 μm. **B** The reflection coefficient of the interdigital transducer was observed with and without the matching network (MN). **C** The streaming velocity in the AFT against input power was showed

The finger electrodes on the PCB are the key element involving coupling radio frequency (RF) signals to the piezoelectric substrate for generating mechanical vibration. The measured IDT width and spacing were 31.0 ± 1.1 μm and 67.6 ± 0.9 μm, respectively, as shown in Fig. 3A, resulting in the SAW wavelength of 197.1 ± 2.4 μm. The working frequency was firstly determined by reading the S_11_ and S_22_ spectrum as shown in Fig. 3B, where the reflection of the IDTs was reduced from -2.5 dB to -53.5 dB, and -2.4 dB to -49.4 dB, respectively, indicating the power transmission was greatly improved by introducing the matching network. The working frequency was set as that produced the minimum reflection, i.e., 19.68 MHz.

The thermal effect in the luminal compartment should be well managed to minimise the impact on the attached hBMECs. The temperature rise inside the compartment was within 0.5 °C under the SAW doses applied during the SAW treatment. As shown in Fig. 3C, measured streaming velocities polystyrene microparticles (size 10 μm) was fully controllable by adjusting the AFT input power.

### Enhancement of BBB permeability in the AFT

**Fig. 4:**
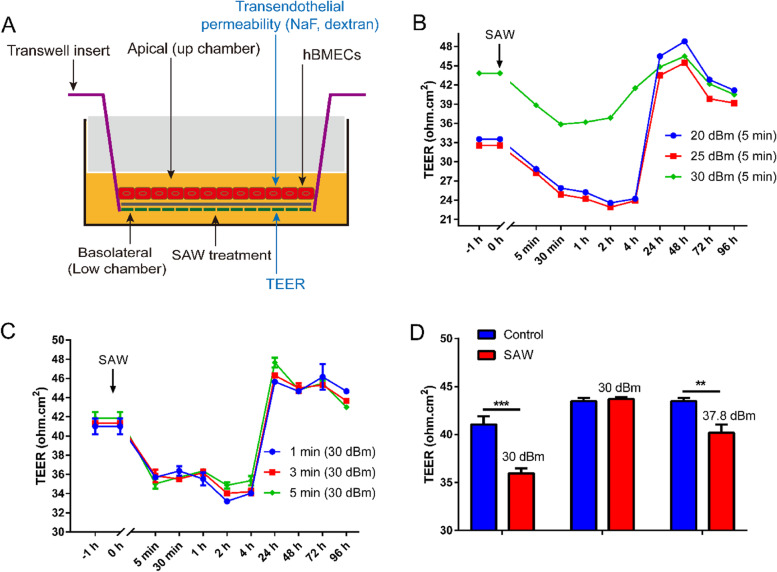
Blood-brain barrier (BBB) permeability was increased under surface acoustic waves (SAW) treatment. **A** A transwell model was constructed to imitate the BBB in vitro. Transwell contains an insert which hanged in the well plate. The bottom of transwell insert was microporous membrane that separates the well plate into two sides. The apical side (up chamber) mimicked the bloodstream, while the basolateral side (low chamber) mimicked the brain. Human brain microvascular endothelial cells (hBMECs) were seed and reached confluence to form the in vitro BBB model. After SAW treatment, the change of transendothelial electrical resistance (TEER) and transendothelial permeability of chemical reagent could be measured. **B** hBMECs were seeded into transwell for 24 h to form the preliminary BBB and then exposed to SAW in 19.68 MHz for 5 min at 20 dBm, 25 dBm and 30 dBm, respectively. The TEER was measured with endohm tissue resistance measurement chamber at the indicated time points. The -1 h represented the initial TEER was measured at 1 h before SAW exposure. Data are representative results of two independent experiments (time point 0 h represented the 24 h incubation of transwell inserts). **C **The BBB was treated with a 19.68 MHz SAW at 30 dBm for 1 min, 3 min, and 5 min, respectively. The TEER was detected at the indicated time points. The data are representative of three independent experiment and displayed as means ± SEM (time point 0 h represented the 24 h incubation of transwell inserts). **D** hBMECs were seeded in transwell, then the BBB was exposed on various power of SAW. The change of TEER was measured immediately. The data is representative of three independent experiment. Data are shown as means ± SEM. **, *p* < 0.01, ***, *p* < 0.001

The transwell insert was coated with type IV collagen protein, which mimicked the basement membrane of the BBB. Then hBMECs were seeded onto transwell insert to form the hBMEC monolayer (Fig. 4A). Usually, the in vitro BBB was formed after 24 h of cell culture, which is characterized by high TEER, normally noted above 30 Ω·cm^2^ in a 24-well transwell plate. After 24 h of cell confluence, the TEER was kept raising because of the cell proliferations, and at about 48 h, a tight BBB was established. The in vitro BBB was treated on AFT with various SAW powers. BBB disruption was immediately noted after SAW stimulations. The TEER of the hBMECs was found to decrease with SAW agitation with power of 20 dBm, 25 dBm, or 30 dBm, which began at the end of SAW stimulation (for 5 min) and then continued up to 4 h (Fig. 4B). Moreover, after overnight incubation, the TEER values of all groups recovered with notable increase at 24 h. The TEER showed a slow reduction after 72 h, which might be due to highly crowded cells consuming the medium nutrition. To explore the relationship between the SAW duration and BBB disruption, the dose of 30 dBm was selected for the following study. The SAW duration was set for 1 min, 3 min, or 5 min, respectively. Similar results were observed that the TEER decreased after SAW agitation for 5 min and TEER value sustained up to 4 h (Fig. 4C). After 24 h, the TEER of hBMECs was resumed completely, reflecting the recovery of the barrier function. To further investigate the susceptibility of BBB to SAW agitation, different status BBB were exposed to SAWs. We observed that a low TEER of BBB was sensitive to SAWs and BBB opening occurred at power of 30 dBm (Fig. 4D). However, but for high TEER of BBB, a higher power of SAW such as 37.8 dBm was required for opening of BBB (Fig. 4D). These results clearly proved that SAW treatment changed the TEER of BBB, but for a tight BBB, a high power SAW was essential for altering the BBB permeability and the BBB was recovered far more rapid.

### SAW opened BBB without affecting cell viability of hBMECs

**Fig. 5:**
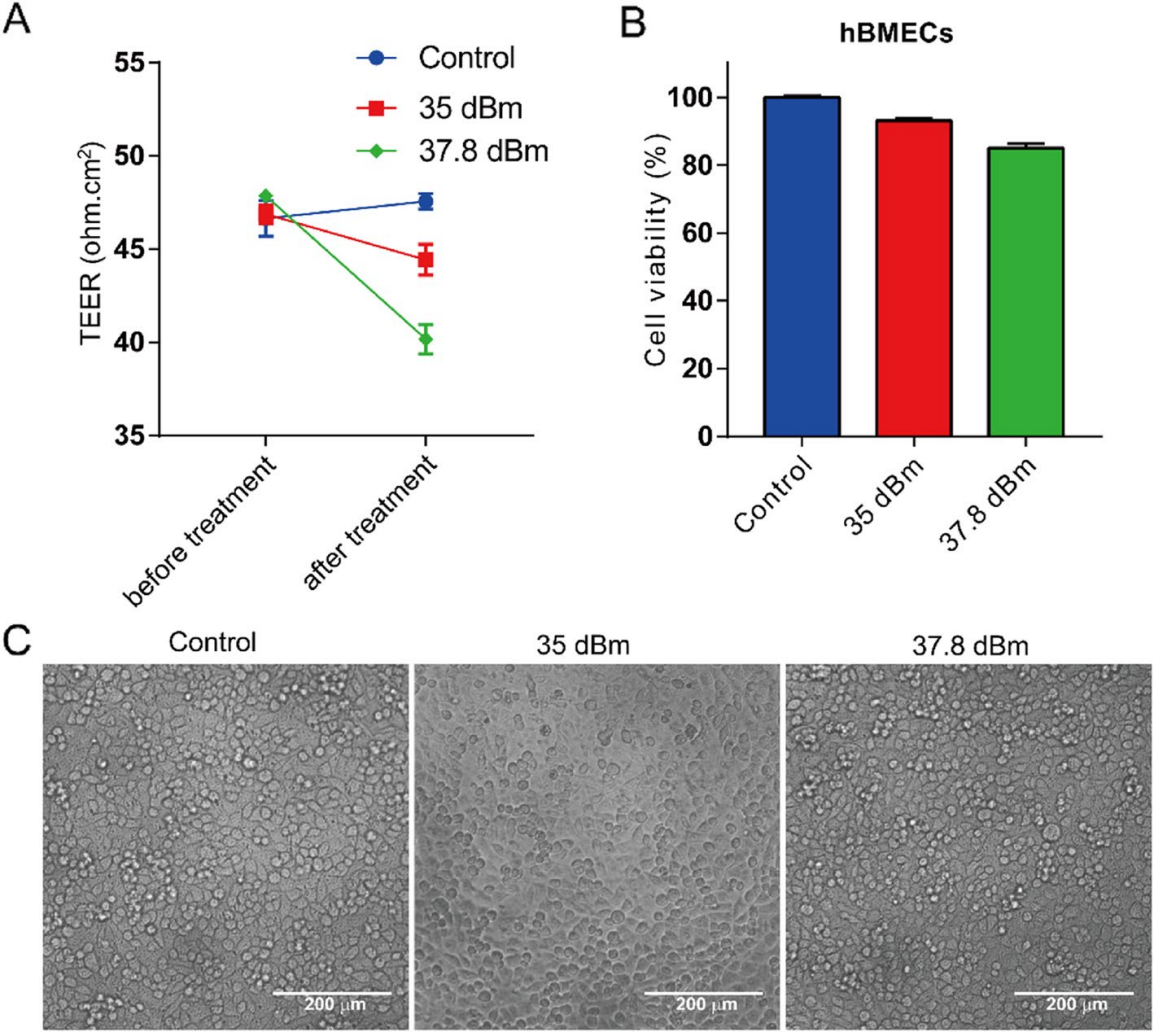
Cell viability of hBMECs in SAW exposure. **A** hBMECs were seeded in transwell for 48 h when the firm barrier formation, then the in vitro BBB was exposed on SAW in 19.68 MHz for 5 min at 35 dBm or 37.8 dBm. TEER was measured before and after SAW treatment. **B** Cell viability in (**A**) was detected with CCK-8 kit. Results are expressed as percentage of cell viability in comparison with control. **C** hBMECs were untreated or exposed to SAW for 5 min at 35 dBm or 37.8 dBm. The bright field images were acquired in an inverted fluorescence microscope. Data are representative results of three individual experiments and shown as means ± SEM. Scale bar was 200 μm

To determine whether SAW exposure would alter the cell viability of hBMECs, a CCK8 kit was applied. The hBMECs were seeded in transwell inserts for 48 h to form the firm BBB, then the inserts were treated with SAW agitations at 35 dBm or 37.8 dBm, for 5 min at a frequency of 19.68 MHz. Results showed that the TEER of hBMECs was dramatically decreased (Fig. 5A), while the cell viability of hBMECs was about 93.1% and 85.1% after 35 dBm or 37.8 dBm SAW treatments (Fig. 5B). Observed using optical microscope, cell morphology of hBMECs turned to be rounder under 35 dBm or 37.8 dBm SAW exposure, but did not show dramatic differences, if compared with that of control group (Fig. 5C). To explore the pathways associated with cell viability loss, a flow cytometry experiment was performed with an Annexin V/PI kit. We found SAW treatment had no impact on apoptosis or necrosis of hBMECs, suggested that cell death was not the leading cause of cell viability loss (Fig. S1 and Table S1). These results suggested SAW stimulation barely affected hBEMCs cell viability, but slightly changed the cell morphology.

### SAW stimulation increased the permeability of chemical drugs cross the BBB

**Fig. 6:**
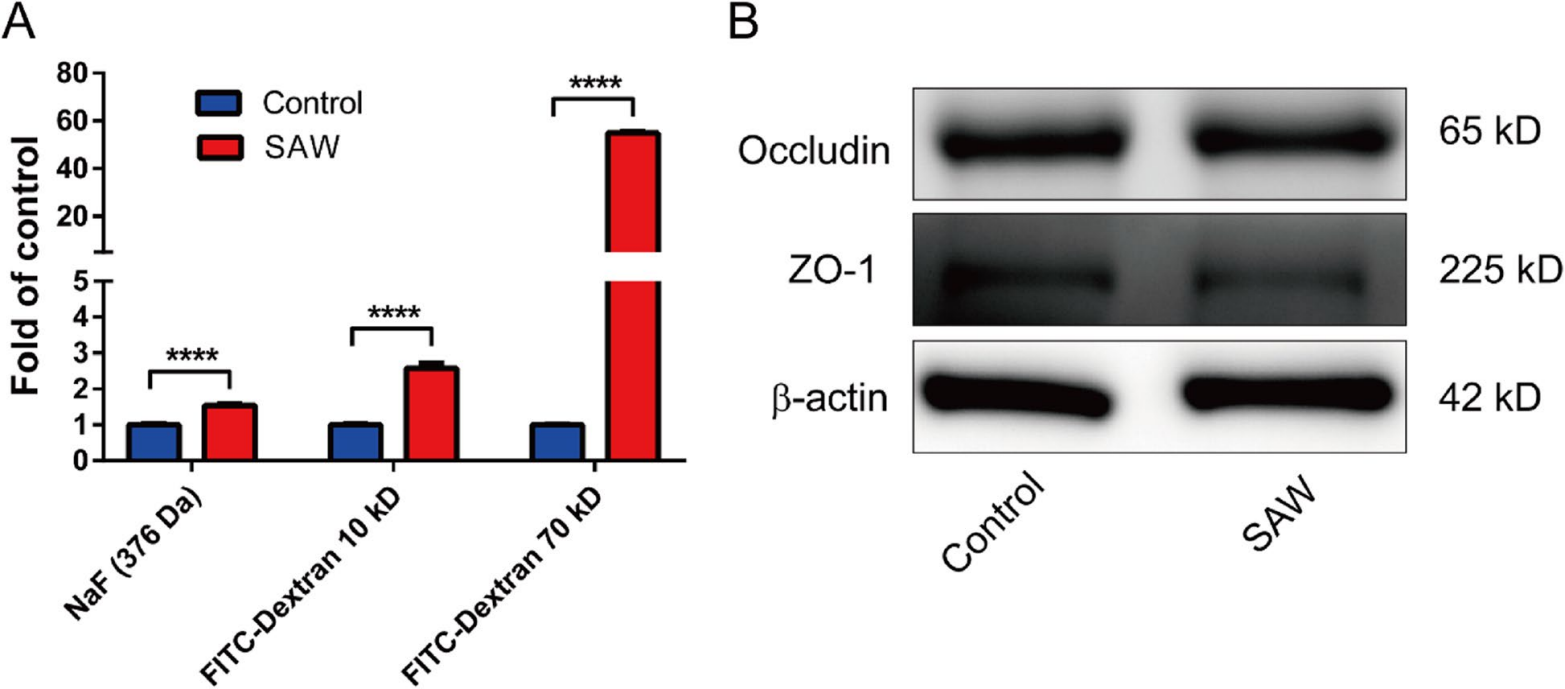
SAW exposure increased transendothelial permeability of hBMECs to chemical drugs. **A** hBMECs were seeded in transwell for 48 h to form firm barrier, and the in vitro BBB was exposed to SAW at 19.68 MHz at 37.8 dBm for 5 min. Then The fluorescein sodium (NaF, 376 Da, 100 μg/ml), FITC-dextran-10 kD (1 mg/ml), or FITC-dextran-70 kD (1 mg/ml) were added to the apical side (up chamber) to access the permeability of in vitro BBB. The ratio of fluorescence intensity in low chamber to up chamber was normalized with control group. **B** Expression of the tight junction proteins Occludin and zonula occludens 1 (ZO-1) were detected with western blotting after SAW treatment (19.68 MHz, 37.8 dBm, 5 min). Representative result of three independent experiments was shown (means ± SEM). ****, *p* < 0.0001

Fluorescein sodium (NaF) and fluorescein-labeled dextran are generally used as indicators to test the BBB permeability [35, 37]. In this study, NaF (376 Da), FITC-dextran-10 kD, and FITC-dextran-70 kD were selected, which represented the small molecule and macromolecular chemical drugs, respectively. To evaluate the transendothelial permeability of BBB to drugs with different molecular weights, the transwell inserts were exposed to SAW (with a frequency of 19.68 MHz) at a power of 37.8 dBm for 5 min, and then NaF or FITC-dextran was added to the upper chamber of inserts as illustrated in Fig. 4A. It was observed that all of NaF, FITC-dextran-10 kD, and FITC-dextran-70 kD in the lower chamber showed an increase of permeation after SAW treatment in AFT device (Fig. 6A). The ratio of fluorescence intensity in lower chamber to upper chamber exhibited about 1.5-, 2.6-, and 54.9-folds increase respectively in NaF, FITC-dextran-10 kD, and FITC-dextran-70 kD group compared with the control group (Fig. 6A). BBB disruption was usually associated with the down regulation of TJ proteins [38]. The expression of Occludin and ZO-1 was determined by western blotting. There were no significant changes observed on ZO-1 and Occludin expression after SAW treatment (Fig. 6B). These data clearly implied that SAW agitation enhanced BBB permeability, and the BBB disruption was independent of the expression regulation of TJ proteins.

### SAW damaged BBB integrity by enlarging the intercellular space of hBMECs

**Fig. 7:**
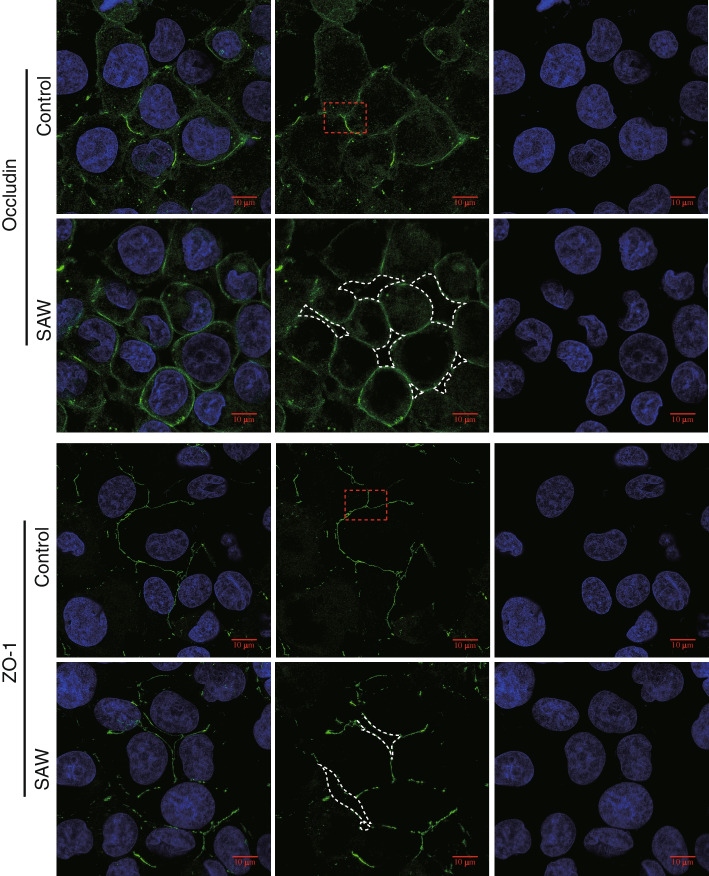
SAW expanded intercellular space of hBMECs. hBMECs were seeded on round slides for 48 h to firm barrier, then exposed on a 19.68 MHz SAW at 37.8 dBm for 5 min. The slides were subjected to immunofluorescence staining. The distribution of tight junction proteins was hybridized with the corresponding anti-Occludin (**A**) or anti-ZO-1 (**B**) antibody. Nuclei was marked with Hochest 33,342 and shown in blue. The representative image of two independent experiments were shown. Scale bar was 10 μm

To further explore the mechanisms that related to SAW induced BBB breakdown, the immunostaining was performed to determine the expression and distribution of TJ proteins on hBMECs. The hBMECs were seeded on slides for 48 h, then the slides were exposed to SAW at 19.68 MHz, 37.8 dBm for 5 min and subjected to immunostaining promptly. Occludin and ZO-1 were continuously expressed in the cell membrane encircling the cells and delineating the cellular borders of hBMECs, while small fraction of the Occludin was also expressed in cytoplasm (Fig. 7Aa, Ba, Fig. S2). As reported, once the BBB was formed in vitro, TJ sealed the intercellular space (red dotted line marking area) maintaining a lower paracellular permeability (Fig. 7Ab, Bb, and Fig. S2). Once exposed to SAW on the AFT device, the interval between adjacent cells (e.g., white dotted line marking area) were unzipped and showed enlarged intercellular space (Fig. 7Ae, Be, and Fig. S2), corresponding to the permeability increase as shown in Fig. 6A. These results demonstrated that the SAW did not affect the expression of TJ proteins but disrupted the TJs connection among hBMECs, which was possibly the main reason resulting in an increased paracellular permeability.

### SAW exposure promoted NaF permeation into zebrafish brain

**Fig. 8:**
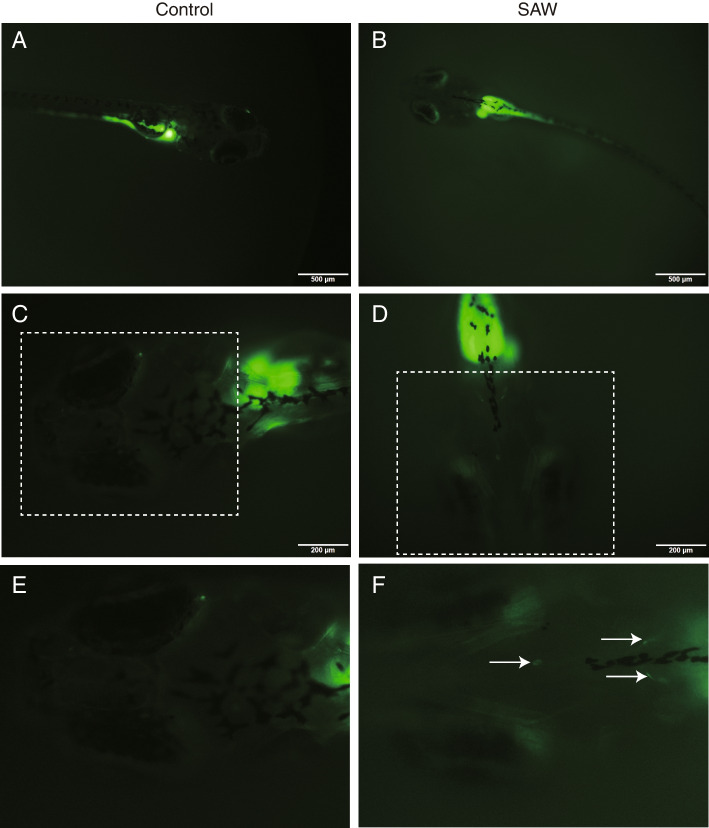
SAW induced fluorescein sodium (NaF) penetrated the brain of zebrafish. 10-day-old zebrafish was maintained in 1 mg/ml NaF solution, and then exposed with SAW (19.68 MHz, 37.8 dBm, 5 min). Control group was maintained in NaF solution without SAW stimulation. After 5 min, the zebrafish was washed in fresh water and observed under a fluorescence microscope. Data are representative results of two individual experiments. e and f were the enlargement of the white dashed box in c and d, respectively. Scale bar was 500 μm in (**A**, **B**) and 200 μm in (**C**, **D**)

For further investigation whether SAW facilitate in vivo drugs delivery, a zebrafish model was applied. The 10-day-old (i.e., 10 days post fertilization) zebrafish were applied because they are clearly visible for the observation of NaF penetration into the brain of fish with transparent body shapes under the fluorescence microscope. The brain areas of zebrafish are generally consisted of four parts. From the anterior to posterior, the zebrafish brain could be dissected to telencephalon, optic tectum, cerebellum and brainstem (Fig. S3) as previously reported [39]. The zebrafish were kept in 1 mg/ml NaF solution with or without SAW stimulation (19.68 MHz, 37.8 dBm) for 5 min, then the zebrafish were observed under the fluorescence microscope immediately. At a low magnification, the full zebrafish were seen, and a strong signal of fluorescence was located in the digestive tract (Fig. 8A, B). After zoomed in, the fluorescence signal was visible in zebrafish brain (Fig. 8C, D, E, F). Particularly, with SAW stimulation, the NaF were penetrated into the zebrafish’s brain (white arrow) and were punctate distributed (Fig. 8F). Unexpectedly, we also discovered an increase permeation of NaF to the eyes of zebrafish after SAW exposure (Fig. 8B). Considering that the eyes contain blood–retinal barrier, which is similar with blood–brain barrier, we assumed that enhancement of drug penetration using SAWs was not only limited to the BBB since the whole body of zebrafish exposed to the SAWs. These results clearly showed that SAW exposure led to an increase of barrier permeability in zebrafish, consequently, the NaF penetrated into the zebrafish brain.

## Discussion

The characteristics of BBB pose a tremendous challenge for the delivery of compounds to the brain parenchyma, leading to a low uptake of biotherapeutics and unsatisfactory treatment outcomes. Among multiple strategies, actively opening the BBB using the ultrasound was demonstrated to improve the efficiency of drug delivery, however, cavitation induced brain damage during treatment was usually unavoidable [40]. In this study, we developed the AFT device that was integrated with the transwell model. We found that the preliminary BBB (24 h) was susceptible to SAW exposure, in which a lower SAW power and less time of SAW stimulation was sufficient to enhance BBB permeability. Tight BBB (48 h) was stable under low power of SAWs, however, with the increase of SAW power in the AFT device, the tight BBB showed an increased permeability. The reason for different sensitivities to SAW exposure might be attributed to the TJ proteins between hBMECs on early BBB were relatively loose and became secure after extra time of hBMECs cultivation. By selecting the appropriate power of SAWs, we confirmed it is a promising method for CNS drugs delivery by reversibly opening the BBB.

We observed the integrity of BBB was disrupted with various SAW power and durations in the AFT device. After a short period of culture, the integrity of BBB was recovered by the cell growth, which showed a reversible and controllable process. In fact, the TJ proteins of BBB were dynamic [41], which might recover to normal after moderate destruction. The BBB in organism is relatively stable and insusceptible. Some cytokines and virus such as interferon and West Nile virus even reinforced the barrier function of BBB [42], while the inflammatory factors and peroxynitrite could damage BBB [37, 43]. The tight BBB did not show the permeability changes until exposure to a higher SAW power on AFT device, indicating that the SAW power should be adjusted according to the TEER of BBB. Indeed, the TEER of firm BBB was found to reduce after 35 or 37.8 dBm SAW exposure, but the cell viability still maintained at 85.1% even a 37.8 dBm SAW exposure. The cell death was not showed significant increase after SAW treatment. To ensure high drug delivery efficiency to CNS, proper parameter of SAW is needed. The form and power of SAW induce moderate openness of BBB, while time interval maintains BBB opening. However, SAW exposure did show morphology changes of the roundness of hBMECs in the AFT device, leading to an increase intercellular space among cells, which might be responsible for the change of BBB permeability.

BBB disruption was usually related to the degradation of TJ proteins. For example, Interleukin 6 disrupted the integrity of BBB via activating ubiquitin–protein ligase E3 and promoting proteasomal mediated ZO-1 degradation [32, 37]. Another study suggested that the degradation of TJ proteins was possibly correlated with autophagy in an experimental autoimmune encephalomyelitis model [44]. Unexpectedly, we found the expression level of Occludin and ZO-1 was almost no visible alterations after SAW exposure in the AFT device, indicating that SAW induced BBB breakdown was not via degradation the TJ proteins. In fact, it is only required a short time for the SAW stimulation on hBMECs (e.g., up to 5 min), it was probably not through the degradation of TJ proteins by proteasome or autophagy route. The mechanism associated BBB disruption after SAW stimulation might be related to the change of cell arrangement. Indeed, we observed the enlargement of intercellular space of the adjacent cells after SAW exposure compared to those of the control group, and we did not see apparent changes of expression of TJ proteins. It is more like unzipping the adjacent cell membranes, which reflected an instantaneous and effective pulling force on the cells. After another 24 h culture, the intercellular space between adjacent cells was disappeared (data not shown), suggested a recovery of BBB. Our results could not rule out the impact of cell death on the integrity of the BBB after SAW treatment. The cell viability and apoptosis results suggested that cell death seemed not the leading cause of BBB opening. SAW induced BBB disruption might because that the SAW mode we were using was Rayleigh wave mode, which has both a longitudinal and a vertically component [45]. The complex forces of SAW agitations were able to pull and open the adjacent cells. Other ways that disrupted BBB were possibly electromagnetic fields, thermal effects, and acoustic streaming [46]. Local excessive heating generated by SAWs might change the state of the cells [47], and cell shapes were believed to affect under periodic acoustic streaming by SAW excitation [48]. More studies are needed to clarify the specific mechanism of SAW induced BBB breakdown.

However, there are also inadequacy results in the current study. The limit of acoustic instrument and the thermogenesis of piezoelectric LiNbO_3_ restricted the high power of SAW device. A semiconductor temperature reducer was used in our study when the BBB exposed to a high power of SAW. The TEER detection and transendothelial cell permeability measurement were only performed at the given time of the experiment and not a real time monitoring process. In this way, the slight inflection of the BBB integrity could not be easily observed. The in vivo trial showed a relatively weak fluorescence signals in zebrafish brain, which might because the AFT device was not fully adapted to the alive and kicking fish. The energy of SAW wave in AFT device has been attenuated after crossing the medium, skin and brain parenchyma to microvessel wall of zebrafish. We observed the penetration of NaF to the eyes of zebrafish, suggesting that SAW of AFT device could also disrupt blood–retinal barrier. The eyes showed a much higher fluorescence intensity than that in the brain. This might because the eyes were not fully covered by the skeleton, and the skin around eyes was so thin, that the SAW was easily accessible to the barrier sites.

## Conclusions

In general, this research applied the SAW device to stimulate the BBB which was not reported before. The SAW platform can be applied as a useful tool that reversibly opens the BBB with minimized damages of the long-term barrier function of BBB, which should be developed as an alternative pathway for CNS drug delivery.

## Supplementary Information

**Additional file 1: Supplementary Fig. 1.** Apoptosis analysis after SAW treatment. hBMECs were seeded in round slides for 48 h when firm barrier formed, then the slides were exposed on a SAW. The apoptosis of hBMECs was measured with Annexin V-AbFluor-647 Apoptosis Detection kit. Data are representative results of two individual experiments. **Supplementary Table 1.** The statistical analysis of Supplementary Fig. 1. **Supplementary Fig. 2.** Three-dimensionalimage of tight junction proteins. hBMECs were seeded in round slides for 48 h when firm barrier formed, then the slides were exposed on a SAW, which was same with Fig. 6. The z axis was selected for 10 μm, and 11 continuous pictures were acquired. The 3-D distribution of cell tight junction proteins was reconstituted and shown. **Supplementary Fig. 3.** A schematic diagram of brain region in 10-day-old zebrafish. The main brain regions include telencephalon, optic tectum, cerebellum and brainstem. The dotted lines only indicate the relative position of each brain region, and the area does not represent the full size of each region. Scale bar was 200 μm.

## Data Availability

The datasets used during the current study are available from the corresponding author on reasonable request.

## Abbreviations

AFT: Acoustofluidic transwell
BBB: Blood–brain barrier
BBBO: Blood–brain barrier opening
CNS: Central nervous system
hBMECs: Human brain microvascular endothelial cells
SAW: Surface acoustic wave
TJs: Tight junctions
TEER: Transendothelial electrical resistance
