# Supplementary material for: Open source board based acoustofluidic transwells for reversible disruption of the blood–brain barrier for therapeutic delivery
Source: Biomater Res. 2023 Jul 15;27:69. doi: 10.1186/s40824-023-00406-6 (PMC10349484; doi:10.1186/s40824-023-00406-6)
Supplement: Supplementary file 1 — Additional file 1: Supplementary Fig. 1. Apoptosis analysis after SAW treatment. hBMECs were seeded in round slides for 48 h when firm barrier formed, then the slides were exposed on a SAW. The apoptosis of hBMECs was measured with Annexin V-AbFluor-647 Apoptosis Detection kit. Data are representative results of two individual experiments. Supplementary Table 1. The statistical analysis of Supplementary Fig. 1. Supplementary Fig. 2. Three-dimensionalimage of tight junction proteins. hBMECs were seeded in round slides for 48 h when firm barrier formed, then the slides were exposed on a SAW, which was same with Fig. 6. The z axis was selected for 10 μm, and 11 continuous pictures were acquired. The 3-D distribution of cell tight junction proteins was reconstituted and shown. Supplementary Fig. 3. A schematic diagram of brain region in 10-day-old zebrafish. The main brain regions include telencephalon, optic tectum, cerebellum and brainstem. The dotted lines only indicate the relative position of each brain region, and the area does not represent the full size of each region. Scale bar was 200 μm. [file 40824_2023_406_MOESM1_ESM.docx]

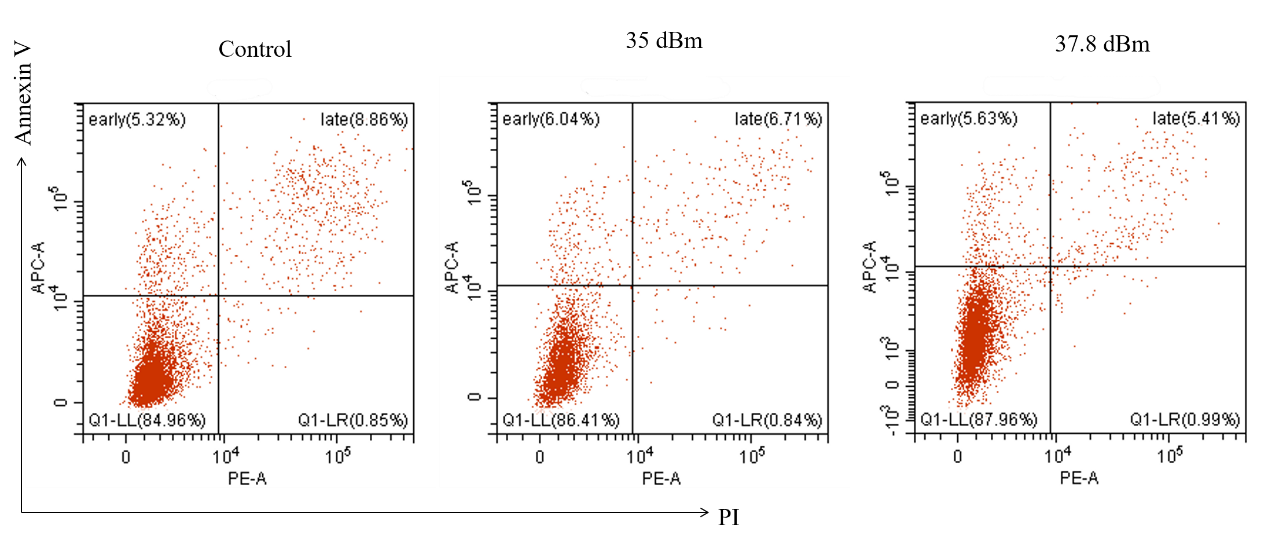


Supplementary Figure 1: Apoptosis analysis after SAW treatment. hBMECs were seeded in round slides for 48 h when firm barrier formed, then the slides were exposed on a SAW (19.68 MHz, 35 dBm or 37.8 dBm, 5 min). The apoptosis of hBMECs was measured with Annexin Ⅴ-AbFluor-647 Apoptosis Detection kit. Data are representative results of two individual experiments.

Supplementary Table 1: The statistical analysis of Supplementary Figure 1.

| Groups | Annexin Ⅴ^+^PI^-^ (%) | Annexin Ⅴ^+^PI^+^ (%) | Annexin Ⅴ^-^PI^+^ (%) |
| --- | --- | --- | --- |
| Control | 4.84±0.34 | 6.31±1.80 | 0.85±0.19 |
| 35 dBm | 5.14±0.94 | 4.06±0.94 | 0.50±0.10 |
| 37.8 dBm | 5.67±0.47 | 5.09±0.82 | 1.39±0.14 |

The results in Supplementary Figure 1 were analysed in Supplementary Table 1. Annexin Ⅴ^+^PI^-^ and Annexin Ⅴ^+^PI^+^ represented early and late apoptosis cell populations, respectively. Annexin Ⅴ^-^PI^+^ could be referred as necrosis cell population as previously described [1, 2]. Data are shown as means±SEM.


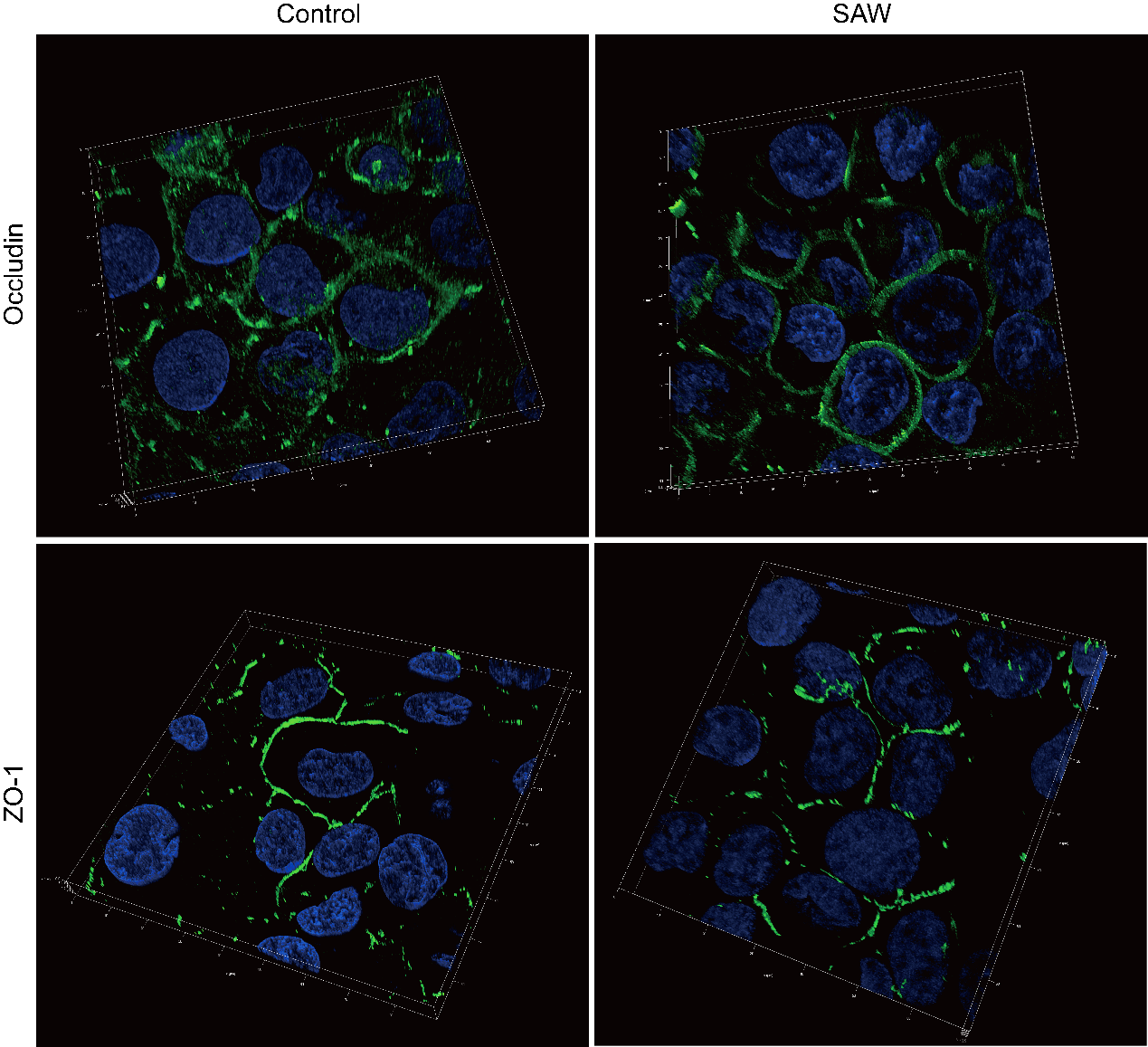


Supplementary Figure 2: Three-dimensional (3-D) image of tight junction proteins. hBMECs were seeded in round slides for 48 h when firm barrier formed, then the slides were exposed on a SAW (19.68 MHz, 37.8 dBm, 5 min), which was same with treatment in Figure 7. The z axis was selected for 10 μm, and 11 continuous pictures were acquired. The 3-D distribution of cell tight junction proteins was reconstituted and shown.


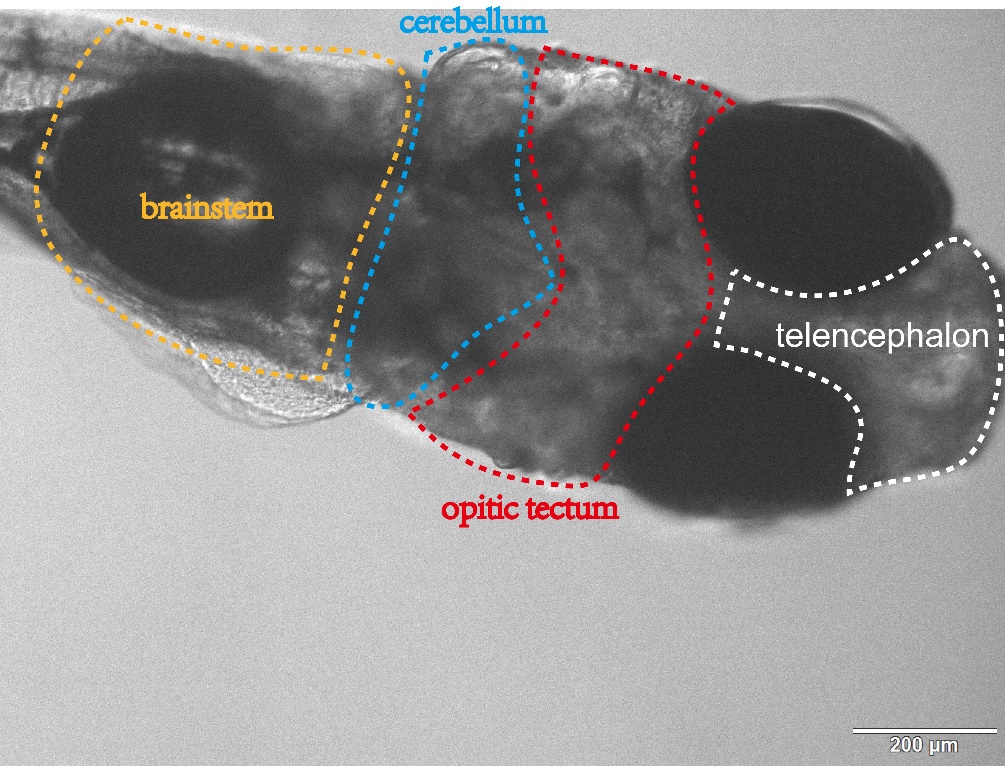


Supplementary Figure 3: A schematic diagram of brain region in 10-day-old zebrafish. The main brain regions include telencephalon, optic tectum, cerebellum and brainstem. The dotted lines only indicate the relative position of each brain region, and the area does not represent the full size of each region. Scale bar was 200 μm.

References

[1] A.L. Edinger, C.B. Thompson, Death by design: apoptosis, necrosis and autophagy, Curr Opin Cell Biol 16(6) (2004) 663-9.

[2] C. Lohmann, A. Muschaweckh, S. Kirschnek, L. Jennen, H. Wagner, G. Häcker, Induction of Tumor Cell Apoptosis or Necrosis by Conditional Expression of Cell Death Proteins: Analysis of Cell Death Pathways and In Vitro Immune Stimulatory Potential 1, The Journal of Immunology 182(8) (2009) 4538-4546.
